# The effect of continuous vs. intermittent protocols during small-sided games in soccer on physiological, physical, technical and tactical performance

**DOI:** 10.3389/fspor.2025.1722658

**Published:** 2025-11-20

**Authors:** Michael C. Rumpf, Johannes Jäger, Matthias Lochmann

**Affiliations:** 1 Department of Sport Science and Sport, Friedrich-Alexander University Erlangen-Nürnberg, Erlangen, Germany; 2 Footballscience.net, Dreieich, Germany; 3 Sport Performance Research Institute New Zealand, AUT-University, Auckland, New Zealand

**Keywords:** small-sided and conditioned games, soccer training, football (soccer), continuous protocol, task constraints

## Abstract

Small-sided games (SSG) are a common part of the soccer practice and coaching regularly utilize continuous (CSSG) or intermittent games (ISSG). Interestingly, players enjoy the CSSG format to a greater extent, whilst experiencing greater individual perceived exertion (RPE) and higher blood lactate concentration. CSSG reported a decrease in maximal and average heart rate and lower time spent in high heart rate zones (>90% maximum heart rate). Players experienced lower physical strain during CSSG. Variables associated with higher external load such as total distance, total number of sprints, and distances in high-intensity speed thresholds (>14.4 km/h) as well as player load and acceleration variables decreased during CSSG. Little research opposes this idea reporting increased physical demands with the identical variables. The technical performances of players in the two different regimes showed also significant differences. The CSSG reported lower technical engagement (decreased ball involvement, number of dribbling and interceptions). However, yet displaying greater technical efficiency (lower percentage of lost balls, greater percentage successful passes). There is no scientific evidence with regards to tactical differences between the CSSG and ISSG. Depending on the time in the season and the level of players, coaches should favor ISSG promoting greater physical and technical development.

## Introduction

Soccer is a popular sport worldwide and its inheriting training method encompasses small-sided games (SSG) (1). Coaches frequently manipulate task constraints such as field dimensions, number of players, and the rules of the game (2) to create desired outcomes. A fundamental component was the game regimen (3) differentiating between types of protocol (continuous vs. intermittent) indicating the nature of these games (4). Initial studies have compared individual bout durations in youth (5) and adults (6, 7) signaling whether short(er) or long(er) bout duration are taxing players' technical, physiological and physical systems to a greater extent. With regard to the technical dimension, variables have been scarcely investigated, and the limited evidence available suggests that bout duration may not substantially influence technical actions or proficiency (6). Contrarily, Clemente et al. (7) reported almost certain large decreases in physical variables such as total distance and running distance comparing the longer bout duration protocol (i.e., 3 × 6 min) to the shorter one (i.e., 6 × 3 min) (7). Very likely moderate and large decreases were also evident in mechanical variables such as total accelerations and total number of decelerations, respectively (7). Furthermore, the authors stated that longer sets increase the perception of effort (RPE) (7). Nevertheless, Koklu et al. (5) demonstrated that intermittent small-sided games (ISSG) and continuous small-sided games (CSSG) are similar in terms of physiological responses (5). However Fanchini et al. (6) showed a close-to-significant (*p* = 0.057) observation for heart rate, with the long(er) 6 min SSG being lower than the 4 min SSG. The authors (6) concluded that the increase in bout duration from 2 to 6 min resulted in a decrease in intensity only between the 4- and 6-min SSG. Consequently, Koklu et al. (6) concluded that the magnitude of changes in heart rate is probably not enough to induce different training adaptations (6).

Training interventions also showed some differences between ISSG and CSSG, implemented in youth (8, 9) and adult (10) players. Both groups maintained (10) or exhibited improvements (8, 9) in neuromuscular performances such as horizontal jump, as well as aerobic (YoYo Intermittent recovery test level 1; YYIRTL1) and anaerobic power (repeated sprint test; RAST) with no significant between-group differences (8). However, it was also mentioned that ISSG can lead to better neuromuscular [i.e., squat jump (10)], sprinting (10), and anaerobic power (average power (W) based on RAST (9), repeated sprint test [Bangsbo modified sprint test (10)] improvements compared to CSSG. Furthermore, the intermittent protocol also seemed to outweigh the continuous format with regards to enhancing the aerobic capacity (YYIRTL1 (9). Consequently, it seems logical to conclude that the ISSG and CSSG will elicit different effects on the physiological and physical performance of soccer players.

In order to gain a deeper understanding of the possible effects during training, it is vital to disentangle the physiological and physical differences between both regimes. Furthermore, expanding the scientific knowledge on technical and tactical performance during ISSG and CSSG would be of particular interest to coaches seeking to optimize training design. However, to date, no scientific evidence compiling, analyzing and summarizing the information regarding the effect of these two regimes on physiological, physical, technical and tactical performance in SSG. Consequently, this review aims to discuss the effects of ISSG and CSSG on the mentioned components drawing on existing literature that has examined these training regimes.

The individual scientific studies engaged male youth (5, 8, 9, 11–13) and adult (14–22) players in different game formats such as 2 vs. 2 (5, 11, 13), 3 vs. 3 (5, 11, 12, 16, 18, 19), 4 vs. 4 (5, 9, 11, 13–16, 19, 22), 5 vs. 5 (12, 17, 20, 21), 6 vs. 6 (13). The game durations were a total of 6 min (5), 9 min (5), 12 min (5, 11, 14, 16, 18, 19, 22), 16 min (8, 21), 18 min (15, 20), 20 min (12), 24 min (13, 17), 25 min (9), 28 min (8), 30 min (9), 35 min (9), and 40 min (9). Protocols involved 3 × 2 min (5), 6 × 2 min (11), 3 × 3 min (5), 3 × 4 min (5, 11, 14, 16, 18, 19, 22), 4 × 4 min (8), 6 × 4 min (17), 4 × 5 min (8, 12), 5 × 5 min (9), 6 × 5 min (9), 7 × 5 min (9), 8 × 5 min (9), 2 × 6 min (11, 14, 16, 19, 22), 3 × 6 min (15, 20), 4 × 6 min (8, 13, 17), 4 × 7 min (8), 2 × 8 min (21), 2 × 10 min (12), 2 × 12 min (17). However, the reader needs to be cognizant for the remaining review that two scientific reference (14, 22) utilized female players (14) and/or both genders (14, 22) and therefore interpretation warrant cautions. Nevertheless, a summary of all variables and their respective change in the two protocols and game formats can be observed in Table 1.

**Table 1:** Effects of Intermittent and continuous protocols.

Reference	Population	Playing format	Protocol	Outcome in variable as per protocol and/or game format
Koklu et al. (5)	*N* = 20, male youth 16.6 years of age, elite academy	2 vs 2 3 vs 3 4 vs 4	**Intermittent:** 3 × 4 min, 2 × 6 min	*Game format:*
			**Continuous:** 1 × 12 min	**Physiological Variables:** 2 vs 2 Blood lactate ↑
Pancar et al. (8)	*N* = 16, male youth 16.5 years of age	4 vs 4	**Intermittent:** 4 × 4 min, 4 × 5 min, 4 × 6 min, 4 × 7 min	*Continuous:***Physiological Variables:** RPE ↑
			**Continuous**: 1 × 16, 1 × 20, 1 × 24, 1 × 28 min	
Daryanoosh et al. (9)	*N* = 16, male youth 19.5 years of age, different teams from Iranian youth league (3rd division)	4 vs 4	**Intermittent:** 5 × 5 min, 6 × 5 min, 7 × 5 min, 8 × 5 min	*Continuous:***Physiological Variables:** RPE ↑, average heart rate ↓
			**Continuous:** 1 × 25 min, 1 × 30 min, 1 × 35 min, 1 × 40 min	
Koklu et al. (11)	*N* = 16, male youth 17.0 years of age	2 vs 2 3 vs 3 4 vs 4	**Intermittent:** 6 × 2 min,3 × 4 min, 2 × 6 min	*Continuous:***Physiological Variables:** RPE ↑, Blood lactate ↑, % max heart rate ↑
			**Continuous:** 1 × 12 min	**Physical Variables:** Total distance, Distance speed zone 0–6.9 km/h, Distance speed zone 7.0–12.9 km/h, Distance speed zone 13.0–17.9 km/h
Alcantara et al. (12)	*N* = 11, male youth 13.0 years of age, academy of professional team	3 vs 3 5 vs 5	**Intermittent:** 4 × 5 min, 2 × 10 min	*Game format:***Physiological Variables:** 3 vs 3 RPE ↑
			**Continuous:** 1 × 20 min	**Technical Variables:** 3 vs 3% successful passes ↑, ball involvement ↑, goal scored ↑
				*Continuous:***Physiological Variables:** RPE ↑
				**Physical Variables:** Total distance ↓
				**Technical Variables:** Ball involvement ↓
Hill-Haas et al. (13)	*N* = 16, male youth 16.2 years of age, top-level domestic competition	2 vs 2 4 vs 4 6 vs 6	**Intermittent:** 4 × 6 min	*Continuous:***Physiological Variables:** RPE ↑, % max heart rate ↑
			**Continuous:** 1 × 24 min	**Physical variables:** sprint activity ratio ↓, number of sprints ↓, distance 13–19.8 km/h ↓
Dios-Alvarez et al. (14)	*N* = 8, female adult 22.9 years of age, semi-professional players	4 vs 4	**Intermittent:** 3 × 4 min, 2 × 6 min	*Continuous:***Physical variables:** Total distance ↓, acceleration ↓
			**Continuous:** 1 × 12 min	
Yücesoy et al. (15)	*N* = 16, female adult 20.1 years of age N=16 male adult 20.7 years of age, national league players	4 vs 4	**Intermittent:** 3 × 6 min	*Continuous:***Physiological Variables:** Average heart rate ↓
			**Continuous:** 1 × 18 min	**Technical Variables:** Pass accuracy ↓, number of dribbling ↓, number of interceptions ↓, shots on goal ↑
Farhani et al. (16)	*N* = 16, male adult 20.7 years of age, semi-professional players	3 vs 3	**Intermittent:** 3 × 4 min, 2 × 6 min	*Continuous:***Physiological Variables:** Mood ↓
			**Continuous:** 1 × 12 min	**Technical Variables:** % successful passes ↑, % successful tackles ↑, % successful duels ↑, % lost balls ↑
Branquinho et al. (17)	*N* = 20, male adult 25.2 years of age, professional players	5 vs 5	**Intermittent:** 6 × 4 min, 4 × 6 min	*Continuous:***Physiological Variables:** Max ↓ & average heart rate ↓
			**Continuous:** 1 × 24 min	**Physical Variables:** Total distance ↓, distance < 14.4 km/h ↓, distance 14.4–19.8 km/h ↓, distance >19.8 km/h ↓
Mulazimoglu et al. (18)	*N* = 12, male adult 20.8 years of age, university players	3 vs 3	**Intermittent:** 3 × 4 min	*Continuous:***Physiological Variables:** average heart rate ↓, time spent > 95% max heart rate ↓
			**Continuous:** 1 × 12 min	**Physical Variables:** Player load ↓
Farhani et al. (19)	*N* = 16, male adult 20.7 years of age, semi-professional players	3 vs 3 4 vs 4	**Intermittent:** 3 × 4 min, 2 × 6 min	*Game format:***Physiological Variables:** 4 vs 4 RPE ↓, Blood lactate ↓
			**Continuous:** 1 × 12 min	*Continuous:***Physiological Variables:** PACES Enjoyment ↑, % max heart rate ↑
				**Technical Variables:** % lost balls ↓, % successful passes ↑, % successful tackles ↑
Branquinho et al. (20)	*N* = 20, male adult 23.9 years of age, semi-professional players	5 vs 5	**Intermittent:** 3 × 6 min	*Continuous:***Physiological Variables:** average heart rate ↓, % max heart rate ↑
			**Continuous:** 1 × 18 min	**Physical Variables:** Distance >19.8 km/h ↓
Casamichana et al. (21)	*N* = 10, male adult 21.3 years of age, 3rd Spanish division	5 vs 5	**Intermittent:** 2 × 8 min	*Continuous:***Physiological Variables:** Time spent > 90% max heart rate ↓
			**Continuous:** 1 × 16 min	
Farhani et al. (22)	*N* = 16, female adult 20.1 years of age *N* = 16 male adult 20.7 years of age, national league players	4 vs 4	**Intermittent:** 3 × 4, 2 × 6 min	*Continuous:* **Physiological Variables:** RPE ↑, Blood lactate ↑, PACES Enjoyment ↑, max heart rate ↑
			**Continuous:** 1 × 12 min	**Technical Variables:** % lost balls ↓, % successful passes ↑, % successful tackles ↑

RPE, Rating of perceived exertion; % = percentage, max = maximum, ↑ = significant increase, ↓ = significant decrease.

## Physiological variables

Interestingly, players feel greater exertion (ratio of perceived exertion, RPE) (8, 9, 11–13, 22) and experience higher amount of blood lactate concentration (11, 22), whilst enjoying the continuous protocol to a greater extend (19, 22) compared to the intermittent one. This could be (at least in parts) due to a decrease in maximal (17, 20) and average heart rate (9, 15, 17, 18, 20) and the time spent in high [>90% (21) and >95% (18) maximum heart rate] heart rate zones in CSSG. Little research opposes this idea. However, it was also mentioned that the mood decreases (16), concomitant with an increase in peak heart rate (22) and greater percentage of maximum heart rate (11, 13, 19) utilizing a continuous protocol. Whether these discrepancies derived from so-called task constraints however remains unresolved. Nevertheless, there seemed to be an effect of number of players (19), number of bout and bout duration (11, 12).

RPE and blood lactate values were significantly lower in the big(ger) format (4 vs. 4) compared to the small(er) one (3 vs. 3) regarding the continuous and the longer intermittent format (19). Furthermore, the small(est) format (2 vs. 2) elicited significant greater lactate concentration compared to any other format (5). Consequently, it does not seem surprising that some variables such as RPE seemed to be affected by player number (23) and bout duration. Indeed, higher RPE during the smaller (3 vs. 3) and continuous (1 × 20 min) format (12) was observed. In addition there seemed to be an influence of the duration and number of bouts as the shorter bout elicited similar blood lactate values, and RPE compared to the continuous protocol, while the medium and long bout duration were significantly different (11).

## Physical variables

The scientific evidence regarding the physical variables suggest that the continuous protocol decreased physical strain in players. More precisely, total distance meters (12, 14, 17), sprint activity ratio (AU) (13), total number of sprints (13), distance < 14.4 km/h distance (17), 13-(13) 14.4–19.8 km/h distance (17), >19.8 km/h distance (17, 20), player load (18), and acceleration (14) decreased. There is little scientific evidence opposing this trend at least partly. More precisely Dios-Alvarez et al. (14) reported greater medium- and high-intensity running and player load and decreased low-intensity running in the 3 × 4 min intermittent format, compared to the 2 × 6 min format.

Interestingly, there also seemed to be evidence that the bout duration has an effect on physical parameters (11). The short(est) bout duration [2- (11) and 4 min (14)] compared to the medium [4 min (11)] and long(est) [6 min (14)] duration displayed greater total distance (11, 14), intensity running in speed zones of 7.0–12.9 km/h (11, 14), 13.0–17.9 km/h (11, 14) and lower distance in speed zone 0–6.9 km/h (11, 14), across all utilized game format and player numbers (2 vs. 2, 3 vs. 3 and 4 vs. 4) (11). Furthermore, not only were the protocols examined, however also time periods within each protocol showing that the speed zone 7–12.9 km/h was also associated with a significant difference in the third period (8–12 min), in this case between continuous format (16 min) and intermittent format of 2 repetitions (2 × 8 min) (21).

## Technical variables

Given the nature that the type of protocol affected the physiological and physical variables it seems natural that the technical performances of players also vary due to the utilized protocols. The CSSG protocol resulted in decreased pass accuracy (15), number of dribbling (15), number of interceptions (15), ball involvement (12), % lost balls (19, 22). On the other hand, the following variables were observed higher in the continuous protocol: percentage (%) successful passes (16, 19, 22), % successful tackles (16, 19, 22), % of successful duels (16), % lost balls (16), shots on goal (15).

Similarly with the physiological and the physical parameters, there also seemed to be a trend with regards to number of players involved. Technical variables reported an increase with lower number of players (3 vs. 3 compared to 4 vs. 4 (19) and 5 vs. 5 (12) with the 3 vs. 3 eliciting greater successful passes (12, 19), ball involvement (12), goal scored (12), shots on target (12) compared to 5 vs. 5 (12).

## Tactical variables

To the authors knowledge there is no scientific evidence with regards to the effect of different types of protocol on the physiological, physical, technical and tactical performance during SSG.

## Conclusion

Depending on individual goals for training sessions coaches can choose CSSG or ISSG to favor specific physiological, physical, and technical demands for players. If the aim is to tax physiological adaptations coaches should chose ISSG. Whilst this regime inherited lower enjoyment and perceived exertion for players important physiological variables are higher. Focusing on the physical development of players, coaches should favor ISSG. Not only with regards to the total external load, but also with regards to decisive variables during soccer games such as high-intensity running as well as sprinting. Mechanical variables such as acceleration and deceleration have not been investigated to a great extent. However, it seems that players experience greater load during ISSG. Therefore, a more systematic investigation of mechanical demands across different SSG protocols is warranted in future research to better understand their contribution to overall physical load and player adaptation. The two different protocols (continuous vs. intermittent) involved during SSG display specific practical implications. For example, during loading cycles for season preparation and/or training days with higher intended physiological and physical loads (i.e., match day +3 and −3), coaches should choose the intermittent protocol. For training days closer to match day and for recovery purposes, the continuous protocol seems to hold greater advantages such as greater enjoyment. Nevertheless, coaches need to consider a relative short continuous bout as players perceive CSSG with greater exertion.

Interestingly, the technical demands of ISSG seemed to be higher compared to CSSG. Players seemed to experience greater ball involvement (e.g., number of dribbling), however, in return experience greater negative technical performance (e.g., unsuccessful passes) in the ISSG. This has direct consequences for daily training. For example, coaches who want to improve players dribbling and passing ability should utilize ISSG to a greater extent compared to CSSG. Furthermore, this should be taken into account for coaches training developing players who require lots of technical involvement. More precisely, greater number of passes, number of dribbling and fewer shots on goal during ISSG elicit more time on the ball for individual players and therefore greater learning experience. Furthermore, players that are physically and biologically less advanced (e.g., lower biological maturity) receive similar involvement with the ball and consequently might remain in the long-term-player process.

While evidence about the effects of ISSG and CSSG on physiological, physical, and technical parameters exists, there remains a gap in the literature regarding their impact on tactical demands. In particular, the influence of different SSG formats on tactical behavior, decision-making processes, spatial-temporal dynamics, and collective team organization has received limited attention. Considering that tactical performance is a critical component of match-play effectiveness, understanding how CSSG and ISSG shape tactical behaviors, such as pressing intensity, positioning, off-ball movements, and team coordination, would provide valuable insights for optimizing training designs. Furthermore, coaches need to be aware that number of players involved in both regimes impact physiological, physical, as well as technical performance of players and it seems that the smaller number of players provoke greater involvement in general. More precisely, applying SSG with lower number of players such as 3 vs. 3 compared to 5 vs. 5 or 7 vs. 7 maximizes technical as well as physical demands of players. Lastly, future research implementing ISSG should pay close attention to the number and between set recovery duration which was reported influential on physiological variables (24). Nevertheless, the investigated physiological variables (e.g., muscle hemoglobin, heart rate) (24) as well as the majority of technical variables (e.g., successful passes, touches in possession) did not differ during bouts suggesting regulating of effort through pacing by the players (25) that coaches should be aware of. Consequently, manipulating player numbers within CSSG and ISSG regimes serves as a tool for coaches to adjust training intensity and emphasize specific performance outcomes according to the objectives of the training session.
